# An Assessment of the Food and Nutrition Security Status of Weaned 7–12 Months Old Children in Rural and Peri-Urban Communities of Gauteng and Limpopo Provinces, South Africa

**DOI:** 10.3390/ijerph14091004

**Published:** 2017-09-01

**Authors:** Sithandiwe Ntila, Muthulisi Siwela, Unathi Kolanisi, Hafiz Abdelgadir, Ashwell Ndhlala

**Affiliations:** 1 School of Agricultural, Earth and Environmental Sciences, University of KwaZulu-Natal, Pietermaritzburg 3201, South Africa; siwelam@ukzn.ac.za (M.S.); kolanisi@ukzn.ac.za (U.K.); 2 Agricultural Research Council: Vegetable and ornamental Plants, Roodeplaat 0001, South Africa; abdelgadirh@arc.agric.za (H.A.); NdhlalaA@arc.agric.za (A.N.)

**Keywords:** food access, dietary diversity, child malnutrition, complementary feeding

## Abstract

This study assessed the food and nutrition security status of children receiving complementary food in rural and peri-urban communities. A group of 106 mothers from Lebowakgomo village and Hammanskraal Township, respectively, participated in the survey. Additionally, six focus group discussions were conducted per study area to assess the mothers’ perceptions about children’s food access. The Children’s Food Insecurity Access Scale (CFIAS) was used to assess the food security status (access) of the children. The Individual Dietary Diversity Score (IDDS) together with the unquantified food consumption frequency survey were used as a proxy measure of the nutritional quality of the children’s diets. The age and weight of the children obtained from the children’s clinic health cards were used to calculate Weight-for-Age Z scores (WAZ) in order to determine the prevalence of underweight children. The findings showed that a large percentage of children were severely food-insecure, 87% and 78%, in rural and peri-urban areas, respectively. Additionally, Lebowakgomo children (23.6%) and Hammanskraal children (17.9%) were severely underweight. Overall, children’s diets in both study areas was characterized by nutrient-deficient complementary foods. Cheaper foods with a longer stomach-filling effect such as white maize meal and sugar were the most commonly purchased and used. Hence, the children consumed very limited amounts of foods rich in proteins, minerals, and vitamins, which significantly increased the risk of their being malnourished.

## 1. Introduction

Food and nutrition security exists when all people at all times have physical, social, and economic access to sufficient, safe, and nutritious food and they are able to adequately utilize and absorb the nutrients in the food in order to be able to live a healthy and active life [1]. In developing countries, the failure to achieve adequate nutrition is aggravated by basic, underlying and immediate factors, which contribute to the causes of child malnutrition [2]. The basic causes of malnutrition, viz., limited access to resources and environmental technology, cultural beliefs, religion and tradition, together with the underlying causes, viz., household food insecurity, inadequate maternal and child care, poor sanitation, inadequate health services, and a lack of information, all contribute to the immediate causes of malnutrition, which are associated with inadequate dietary intake and disease related factors. 

During the first two years of life, children are prone to growth faltering and micronutrient deficiencies. The nutritional vulnerability in this period is due to poor breastfeeding and complementary feeding practices coupled with high rates of infectious diseases [3,4]. Exclusive breastfeeding is sufficient for the first six months of life; however, after six months, exclusive breastfeeding or other forms of breast milk substitute alone are not sufficient to meet the nutritional requirements of a growing child [5]. Therefore, the World Health Organization (WHO) recommends the introduction of safe and nutritious complementary foods to children after six months of life. Unfortunately, regardless of the WHO recommendation, and the mandate of Section 28 (1) of the South African constitution, which states that every child has a right to adequate food [6], most households in South Africa have been reported lacking special food products for children [7], thereby depriving them their basic right to proper food.

Malnutrition is the leading cause of death during childhood, resulting in more than 33% of child deaths worldwide [8]. In Sub-Saharan Africa, there was an increase in the percentage of underweight children from 11.7% to 13.5% between 1990 and 2010 [9]. In 2011, 40% of the children less than five years of age living in Sub-Saharan Africa were stunted [10]. The South African National Health and Nutrition Examination Survey (SANHNES) conducted by the Human Sciences Research Council (HSRC) revealed that poor micronutrient status was common among South African children who are under 5 years [11]. The survey found that, at the national level, vitamin A deficiency prevalence was 43.6%, anemia prevalence was 10.7%, iron depletion was 8.1%, and iron deficiency anemia was 1.9%.

The South African Department of Health reported that 17%, 6%, and 3% of 1–5 years old children in Gauteng province were stunted, underweight, and wasted, respectively, on the other hand, 24%, 12%, and 4% of 1–5 years old children in Limpopo province were stunted, underweight, and wasting, respectively [12]. In addition, a high percentage of 1–5 years old children in Limpopo province were deficient in vitamin A (76%) and iron (14%) compared to Gauteng whose 1–5 years children were deficient in vitamin A (65%) and iron (10%) [12]. Studies conducted in Gauteng province reported that 18% of preschool children were stunted [13] and the prevalence of overweight and stunting were 14.1% and 16.5%, respectively [14]. In Vhembe district, Limpopo province, a number of studies assessed the nutritional status of 3–5 years old children. For instance, 24.4% of the 3–5 years old children were found stunted [15], while the prevalence of wasting, stunting, and underweight was reported to be 1.4%, 18.6% and 0.3%, respectively and the prevalence of zinc deficiency and anemia was 42.6% and 28.5%, respectively [16].

Several studies have assessed the food and nutrition security status of children, but the focus has been on the age ranges 1–5 years and 3–5 years. There is limited or no reported data on the food access and nutritional status of children aged 7–12 months, yet, as stated earlier, it is a critical period because complementary feeding is generally initiated within this period. More so, food and nutrition insecurity is often misperceived as applicable to rural communities. Baseline data about the food and nutrition security status of children in the two study areas was deemed important as it can guide nutrition interventions targeting children aged 7–12 months. Therefore, the study assessed the food and nutrition security and the nutritional status of children aged 7–12 months in rural and peri-urban communities of Lebowakgomo, Limpopo province, and Hammanskraal, Gauteng province, respectively.

## 2. Methodology

### 2.1. Research Technique and Sampling Technique

Quantitative and qualitative research methods were used to collect the data. The two methods were used in order to answer research questions that a single methodology cannot answer [17]. This methodology was found appropriate because the study aimed to find answers for a variety of questions, which included socio-economic, psychological, and technical questions. 

The questionnaire was administered to 212 caregivers (106 in Lebowakgomo and 106 in Hammanskraal) who were recruited using purposive sampling. Purposive sampling is the deliberate choice of an informant due to the qualities the informant possesses [18,19]. The criteria for including participants in the study was that they were mothers of children aged 7–12 months, were easily accessible and willing to participate in the study. Six focus group discussions (FGDs) were conducted per study area, the participants of the focus group discussion were recruited on a voluntary basis from the survey participants; each group was comprised of 12 mothers. 

### 2.2. Description of Study Areas and Gaining Entry to the Communities

The study was conducted in two separate communities that are both of Pedi ethnic culture. The two communities are located in Stinkwater, peri-urban Hammanskraal, in the Gauteng province (25°23′59.99″ S: 28°16′60.00″ E), and Ga-Mphahlele village, rural Lebowakgomo, in the Limpopo province (24°18′0.83″ S: 29°32′33.61″ E), South Africa. Hammanskraal is located in the northern periphery of Region 2 of the City of Tshwane Metropolitan Municipality. The northern periphery is characterized by low density settlements with concentrations of subsidized housing and informal settlements [20]; although the area is urban in character, it is not integrated with the larger urban environment of the metropolitan area. While Lebowakgomo is located in Lepelle–Nkumpi local municipality, the Lepelle–Nkumpi local municipality is pre-dominantly rural and 95% of its land falls under the jurisdiction of traditional authorities [21]. 

In order to gain entry into the communities, viz., Ga-Mphahlele village in Lebowakgomo, Limpopo province, and Stinkwater Township in Hammanskraal, Gauteng province, the researcher arranged meetings with community councillors from both study areas to seek permission to conduct the study. The researcher was attached to a community leader who acted as a gatekeeper and assisted with the organization of venues for the meetings. 

### 2.3. Data Collection Procedure

#### 2.3.1. Survey

The questionnaire was made up of three sections that, respectively, (1) enquired about mothers’ and children’s demographic data, (2) assessed the food security status of children, and (3) assessed nutritional quality of children’s diet and the prevalence of underweight children.

##### Section 1: Demographic Data

This section captured the general demographic profile of the children and the socio-demographic profile of the caregivers.

##### Section 2: Food Security Assessment

The Children Food Insecurity Access Scale (CFIAS) was used to assess the food security status (access) of children aged 7–12 months. The Household Food Insecurity Access Scale (HFIAS) [22] was adjusted to accommodate children, resulting in the CFIAS. HFIAS measures three domains of food insecurity (access), viz., anxiety and uncertainty about food supply, insufficient food quality, and insufficient food quantity, the CFIAS used in this study measured the same aspects but at an individual level. This approach was found appropriate because children receiving complementary food have unique and individual-level food access experiences influenced by their age and developmental stage. The CFIAS consisted of nine ‘occurrence questions’ that represent a generally increasing level of severity of food insecurity (access) and nine ‘frequency-of-occurrence questions’ that were asked as follow-up to each occurrence question to determine how often the condition occurred. Mothers were requested to recall whether they had experienced anxiety and uncertainty about food supply or insufficient quality and quantity of food consumption for their children using a 30-day recall. The mothers were first asked an occurrence question in relation to whether the condition in question had happened in the previous four weeks. For those mothers who answered ‘yes’ to an occurrence question, a frequency-of-occurrence question was asked to determine whether the condition had happened rarely (once or twice), sometimes (three to ten times), or often (more than ten times) in the previous four weeks. The frequency-of-occurrence question was skipped for those mothers who reported that the condition described in the corresponding occurrence question had not been experienced in the past 30 days.

##### Section 3: Nutritional Status Assessment

The Individual Dietary Diversity Score (IDDS) together with the unquantified food consumption frequency survey were used as a proxy measure of the nutritional quality of children’s diet, this method has been used successfully previously [23]. Mothers were asked to recall all the foods consumed by their children the previous day, inside and outside home in the past 24 h. The mothers were asked a series of ‘yes’ or ‘no’ questions. In addition, the mothers were asked to recall how frequently each food group was consumed by their children.

In order to calculate anthropometric indices, which were useful in determining the nutritional status of children (prevalence of underweight children), the age and weight of the children were obtained from the children’s health cards. Mothers were requested to bring their child’s health card on the day of the survey, and, with the mother’s consent, trained field workers assisted with the recording of the child’s weight and age from the health card on the survey questionnaire.

#### 2.3.2. Focus Group Discussions

Six focus group discussions (FGDs) were conducted per study area to assess the perceptions of mothers about access to food by children. The FGDs were conducted following a focus group discussion guide; they were facilitated by field workers who were fluent in *Sepedi*, the local language in the two study areas. The researcher requested consent from mothers to use a digital video camera to record the discussions of each group, the recorded data were transcribed into text. The transcribed text, together with handwritten notes, were used to generate the main findings of the FGDs.

### 2.4. Data Analysis

The responses from the CFIAS were captured on the Statistical Package for Social Sciences (SPSS, International Business Machines Corporation, Armonk, NY, USA), version 21 for Windows, and three types of indicators were calculated, viz., children’s food insecurity access-related conditions, children’s food insecurity access-related domains, and children’s food insecurity access prevalence. The correlation between the calculated indicators and mothers’ socio-demographic characteristics was determined; *p* < 0.05 was considered statistically significant. SPSS was used to compute descriptive statistics—mainly the frequency of children consuming different food groups and food groups consumed the past 24 h. WHO Anthro version 3.2.2 for Personal Computers Software using the Nutritional Survey (NS) module was used to record the anthropometric data and calculate anthropometric indices of the children. The weight and age data set was used to calculate Weight-for-Age Z scores (WAZ) using the software. The software uses Z-score reference points, which were adopted by the WHO from the National Centre for Health Statistics (NCHS)-based growth curves. The prevalence of underweight was classified into two categories, namely, severely underweight and underweight. In this study, the index WAZ was only calculated because health officials in the study areas generally do not measure the height of children due to lack of resources. Thus, the other two popular anthropometric indices, viz., Height-for-Age Z scores (HAZ) and Weight-for-Height Z scores (WHZ) could not be calculated.

### 2.5. Ethical Approval

Ethical approval to conduct the study was obtained from the University of KwaZulu-Natal, Humanities and Social Science Research Ethics Committee (HSS/1244/0150). Additionally, approval to conduct the study in Lebowakgomo was obtained from Lebowakgomo Municipality and in Hammanskraal from Tshwane Municipality.

## 3. Results

### 3.1. Demographic Profile of Caregivers and Their Children

The demographic profile of mothers and their children is shown in Table 1. Approximately 87.7% of Hammanskraal mothers and 52% of Lebowakgomo mothers had completed secondary and post-secondary education. The mothers also indicated that health care centres were the major providers of children’s nutrition education in Hammanskraal (47.2%) and Lebowakgomo (65.7%). Additionally, FGDs indicated that government child grants were the main source of income for mothers in both study areas, and agricultural activities were used as coping strategies during periods of financial stress (not tabulated).

### 3.2. Children’s Food Insecurity Access-Related Conditions, Domains and Prevalence

**Figure 1:**
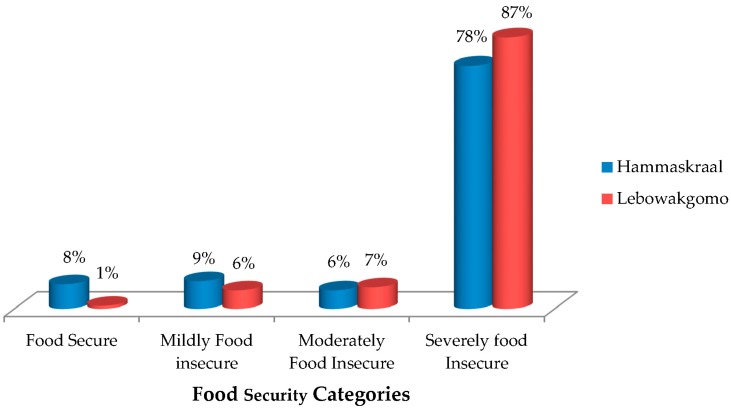
Children’s food security categories as determined by the Children Food Insecurity Access Scale (CFIAS).

Mothers from both study areas experienced food insecurity access-related conditions. In Hammanskraal, mothers were anxious and uncertain about food supply for their children (68.5%) and fed them inadequately, with respect to both quality (72.9%) and quantity (58.6%) (not tabulated). On the other hand, Lebowakgomo mothers were also anxious and uncertain about food supply for their children (87.2%) and fed them inadequately, in terms of both quality (83.4%) and quantity (70.7%) (not tabulated). Consequently, high levels of food insecurity (access) were observed in both study areas (Figure 1). The decrease in household per capita income and limited utilization of agricultural produce when preparing complementary foods increased the level of anxiety and uncertainty about supply of adequate quality food (*p* < 0.05) (not tabulated).

### 3.3. Food Consumption Frequency, Individual Diet Diversity Score (IDDS), and Weight-for-Age (WAZ) as Measures of Nutritional Quality of Children’s Diet and Nutritional Status of the Children

**Figure 2:**
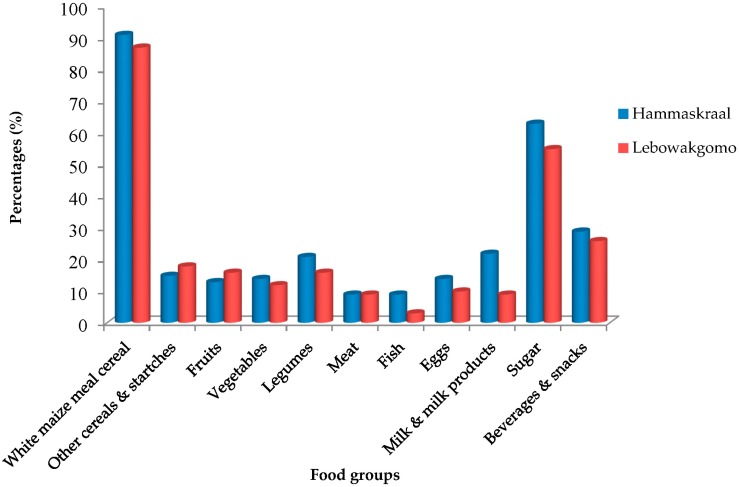
Food groups consumed by children determined using a 24 h recall period.

Mothers acknowledged that various food groups were rarely consumed by the children (Table 2). According to the FGDs, the economic access to complementary foods was a major challenge for mothers in both study areas (not tabulated); consequently, the majority of the children had not consumed various food groups in the previous 24 h (Figure 2). Additionally, FGDs revealed that household food baskets in both study areas were composed of energy-rich foods as they were perceived to be more affordable and filling compared to fruits, vegetables, and meat products, which were perceived to be more expensive (not tabulated). Mothers further indicated in the FGDs that, in order to prepare complementary foods, ingredients were sourced from the household’s food basket. Moreover, soft porridge prepared with white maize meal was the most common complementary food given to children during any time of the day; other ingredients such as sugar, milk powder (powdered formula milk or tea whiteners), margarine, or peanut butter were added to white maize soft porridge to ‘enhance taste’. Stiff porridge prepared with white maize meal together with household relish of the day or with diluted liquid milk were usually given to children as supper, and savoury maize snacks, sweets, juice, and tea were consumed in-between the main meals. Poor food consumption patterns could account for the prevalence of underweight in Hammanskraal and Lebowakgomo children, which can be seen from the results in Table 3. 

## 4. Discussion

The high rate of unemployment and limited utilization of household produced agricultural resources when preparing complementary foods resulted to mothers being more anxious and uncertain about food supply. Consequently, children were not fed adequately. Similarly, a previous study found that limited production coupled with low income increased the tendency of households to pursue diets (including children’s diets) that were inadequate in both quantity and quality [24]. The argument made by Tshabalala (2014) [7] that households in rural South Africa do not purchase special food products for their children is also evident in the findings of this study. Regardless of geographical location, children in this study were generally fed monotonous diets, which were based mainly on starchy and sugary foods, such as soft porridge, sugar, concentrated juice, and tea. White maize meal formed a starch base of almost all the complementary meals given to children during breakfast, lunch, and supper. Similarly, other studies have found white maize to be the dominant base ingredient for complementary foods [23,25,26]. 

Unfortunately, white maize is high in starch and limited in micronutrients [25]. As a result, complementary foods made with white maize as the main or sole solid ingredient, such as soft maize porridge is generally adequate in energy, fibre, and B-vitamins, but deficient in protein and minerals such as iron, calcium, and zinc [27,28]. Although mothers in the current study indicated that other ingredients such as sugar, milk powder, margarine, or peanut butter were added to white maize soft porridge, the porridge may still be nutritional-deficient, as the nutrients may be reduced by overcooking and overdilution of ingredients. The predominant consumption of cereal grain foods and food products rich in energy such as sugar, sweetened tea, and concentrated juice, and limited consumption of fortified commercial foods, fruits, vegetables, and meat products likely increases the risk of micronutrient deficiencies. The consumption of fruits and vegetables has been associated with reduced risk of several chronic diseases later in life [29]. The Child Health Study Group recommends that caregivers gradually include a variety of fruits and vegetables in the diets of children because good health begins at childhood and children are sensitive to malnutrition [30]. In addition, meat has been recommended for children because it is a major source of bioavailable nutrients, including zinc and iron [31,32]. 

The inadequacy of quality food for children observed in this study could account for the varying, generally poor WAZ scores, which were observed in different age groups of both male and female children from both study areas. Lebowakgomo mothers were more anxious and uncertain about food supply and fed their children inadequately with respect to both quality and quantity compared to Hammansraal mothers. Consequently, a high prevalence of underweight and severely underweight children was observed in Lebowakgomo. Although a majority of the mothers from both study areas had received secondary education and had access to a credible provider of nutrition education, children were not fed adequately. Therefore, these findings suggest that the likelihood of children being fed adequately does not necessarily increase with the level of education or provider of children’s nutrition education.

From this study, it is evident that food and nutrition insecurity is not only applicable to rural communities as misperceived; it is also a reality in peri-urban communities. Thus, sustainable interventions aimed at addressing household food and nutrition insecurity are recommended in both rural and peri-urban communities with similar socio-econmic status, focus should be placed on improving basic factors (access to resources and environmental technology), underlying factors (maternal and child care, sanitation, health services, and nutrition education) and immediate factors (physical and economic access to nutrient rich foods). This study was conducted in only two Pedi communities of South African using non-random sampling with extremely small sample size. Therefore, the study findings cannot be used to draw conclusions (generalization) for rural and peri-urban populations of wider geographical regions. Rather, this is an exploratory and descriptive study that provides some evidence of the situation in a defined, very localized geographic area. Further, food and nutrition security is complex and multidimensional with a range of factors that impact food access, availability, utilisation, and stability. Thus, there is a need to integrate various tools when measuring food and nutrition security in order to account for all factors. Unfortunately, due to limited financial resources, this study used only four of the several tools available—the CFIAS, IDDS, unquantified food consumption frequency survey, and antropomentric indices (WAZ).

## 5. Conclusions

High rates of severely food-insecure (access) children were observed in both study areas (Hammanskraal and Lebowakgomo), this contributed to the prevalence of underweight and severely underweight children. Overall, the children’s diets (complementary foods) were characterised by inadequacy in both quantity and quality. These findings provide insight for developing comprehensive and user-friendly food consumption guidelines for these population groups and other population groups of similar socio-economic status. Strategies should be developed to address inappropriate feeding practices through the implementation of programmes aimed at capacitating households with knowledge on dietary diversity, which promote locally available, nutritious, and safe complementary foods.

## Figures and Tables

**Table 1:** Demographic profile of mothers and their children in Hammanskraal (Gauteng) and Lebowakgomo (Limpopo).

Variables	Hammanskraal	Lebowakgomo
**Age of Children**		
7–8 months	50.0	75.5
9–10 months	30.2	15.1
11–12 months	19.8	9.4
**Gender of children**		
Female	58.5	47.2
Male	41.5	52.8
**Age of mothers**		
17–25 years	66	19.8
26–35 years	26.4	73.6
36–45 years	4.7	6.6
46–55 years	2.8	0
**Marital status**		
Single	88.7	90.6
Married	11.3	6.6
Widowed	0	2.8
Divorced	0	0
**Level of education**		
No formal education	3.8	26.4
Primary	8.5	21.7
Secondary	78.3	45.4
Tertiary	9.4	6.6
**Employment status**		
Employed full time	0.9	2.8
Employed part-time	7.5	4.7
Unemployed	91.5	92.5
**Household income per month**		
Below R800	78.3	81.1
R801–R1500	14.2	15.1
R1501–R3500	3.8	1.9
Above R3500	3.8	1.9
**Vegetable garden**		
Yes	2.8	57.5
No	97.2	42.5
**Fruit production**		
Yes	31.1	63.2
No	68.9	36.8
**Livestock**		
Yes	3.8	48.5
No	96.2	51.5
**Nutrition education**		
Radio	5.7	7.1
TV	10.4	2.9
Public health facilities	47.2	65.7
Adult in household	8.5	22.9
None	28.2	1.4

**Table 2:** The usual intake of food items by 7–12 months children as determined by an unquantified food frequency questionnaire (%) administered in two communities (Hammanskraal and Lebowakgomo).

Food Items	Most Days	Once a Week	Seldom	Never
**Cereals/Starches**				
Bread	23.6 ^a^ (5.7) ^b^	7.5 (2.8)	10.4 (6.6)	57.5 (84.9)
Maize meal porridge-Soft	86.8 (88.7)	4.7 (4.7)	3.8 (4.7)	3.8 (1.9)
Maize meal porridge-Stiff	25.5 (25.5)	8.5 (11.3)	17.9 (21.7)	47.2 (41.5)
Maize meal porridge-Fermented	17.0 (29.2)	10.4 (12.3)	9.4 (17.9)	62.3 (40.6)
Cooked porridge other than maize meal	34.0 (45.3)	10.4 (8.5)	5.7 (10.4)	49.1 (35.8)
Infant Cereal	29.2 (4.7)	11.3 (0)	7.5 (6.6)	50.9 (88.7)
Rice	14.2 (0)	16 (0)	3.8 (0)	65.1(0)
Potato	41.5 (13.2)	17.9 (41.5)	6.6 (8.5)	33 (36.8)
**Dairy products**				
Fresh milk	16 (0)	8.5 (0)	8.5 (3.8)	66 (96.2)
Milk powder	32.1 (15.1)	11.3 (8.5)	5.7 (11.3)	50 (65.1)
Yoghurt	26.4 (0)	11.3 (0)	19.8 (15.1)	41.5 (84.9)
**Animal foods**				
Red Meat	1.9 (6.6)	11.3 (0)	1.9 (2.8)	84 (90.6)
Chicken	11.3 (2.8)	6.6 (0)	0.9 (3.8)	80.2 (93.4)
Fish	8.5 (0)	7.5 (0.9)	6.6 (6.6)	76.4 (92.5)
Eggs	21.7 (11.3)	2.8 (7.5)	9.4 (0.9)	65.1 (80.2)
**Legumes**				
Beans	1.9 (8.5)	8.5 ( 6.6)	8.5 (17.0)	80.2 (67.9)
Peanut butter	41.5 (17.0)	10.4 (1.9)	10.4 (13.3)	36.8 (68.9)
**Vegetables**				
Butternut	21.7 (11.3)	15.1 (6.6)	16 (16.0)	46.2 (66.0)
Carrots	17 (3.8)	9.4 (2.8)	6.6 (6.6)	66.0 (86.8)
Dark-green leafy vegetables	6.6 (27.4)	7.5 (9.4)	5.7 (16.0)	79.2 (47.2)
Cabbage	8.5 (12.3)	12.3 (5.7)	2.8 (7.5)	75.5 (74.5)
Tomato	16 (31.1)	2.8 (9.4)	2.8 (10.4)	77.4 (62.3)
**Fruits**				
Apple	12.3 (6.6)	8.5 (0)	6.6 (13.2)	71.7 (80.2)
Banana	16.0 (31.1)	4.7 (9.4)	12.3 (17.9)	66 (41.5)
Orange	17.9 (22.6)	9.4 (10.4)	9.4 (12.3)	62.3 (54.7)
**Miscellaneous**				
Sugar	54.7 (39.6)	18.9 (17.9)	6.6 (10.4)	18.9 (32.1)
Biscuits	24.5 (16)	11.3 (12.3)	22.6 (18.9)	40.6 (52.8)
Sweets	20.8 (22.6)	12.3 (11.3)	21.7 (16.0)	44.3 (50.0)
Savoury snacks	13.2 (14.2)	21.7 (16.0)	19.8 (14.2)	45.3 (55.7)
Carbonated drinks	11.3 (0.9)	10.4 (0)	16.0 (22.6)	61.3 (76.4)
Concentrated Juice	67.9 (58.0)	12.3 (12.3)	5.7 (10.4)	13.2 (22.6)
Tea	67 (54.7)	5.7 (6.6)	7.5 (14.2)	18.9 (24.5)
Coffee	3.8 (0.9)	1.9 (1.9)	1.9 (97.2)	91.5 (97.2)

^a^ Hammanskraal; ^b^ Lebowakgomo.

**Table 3:** The prevalence of underweight in Hammanskraal and Lebowakgomo determined by Weight for Age (Z-score).

Age Groups (Months)	*n*	Severely Underweight (<−3SD)%	Underweight (<−2SD)%	WAZ (SD)
7–8	53 ^a^; 80 ^b^	13.2; 22.5	34.0; 38.8	−1.04 (1.81); −1.12 (2.32)
9–10	32; 16	31.3; 31.6	34.4; 43.8	−0.95 (2.35); −1.99 (2.09)
11–12	21; 10	9.5; 20.0	19.0; 30.0	−0.95 (1.14); −1.09 (1.99)
7–12	106; 106	17.9; 23.6	31.1; 38.7	−0.99 (1.9); −1.25 (2.26)

^a^ Hammanskraal; ^b^ Lebowakgomo.
